# Phenotypical predictors of pregnancy-related restless legs syndrome and their association with basal ganglia and the limbic circuits

**DOI:** 10.1038/s41598-021-89360-8

**Published:** 2021-05-11

**Authors:** Natalia Chechko, Jeremy Lefort-Besnard, Tamme W. Goecke, Markus Frensch, Patricia Schnakenberg, Susanne Stickel, Danilo Bzdok

**Affiliations:** 1 Department of Psychiatry, Psychotherapy, and Psychosomatics, RWTH Aachen University, Pauwelsstrasse 30, 52070 Aachen, Germany; 2 Jülich Aachen Research Alliance (JARA) — Translational Brain Medicine, Pauwelsstrasse 30, 52070 Aachen, Germany; 3 Parietal Team, INRIA, CEA, Université Paris-Saclay, 3 rue Joliot-Curie, 91192 Gif-sur-Yvette, France; 4 Department of Biomedical Engineering, Faculty of Medicine, Montreal Neurological Institute, McGill University, 845 Sherbrooke St W, Montreal, QC Canada; 5 Mila - Quebec Artificial Intelligence Institute, 6666 St Urbain St, Montreal, QC Canada; 6 Institute of Neuroscience and Medicine, Brain & Behaviour (INM-7), Research Centre Jülich, Wilhelm-Johnen-Strasse, 52425 Jülich, Germany; 7 Department of Obstetrics, RoMed Hospital Rosenheim, Pettenkoferstraße 10, 83022 Rosenheim, Germany; 8 Department of Gynaecology and Obstetrics, RWTH Aachen University, Pauwelsstrasse 30, 52070 Aachen, Germany; 9 Klinikum Mutterhaus Der Borromäerinnen gGmbH, Abteilung Für Gynäkologie U. Geburtshilfe, Feldstraße 16, 54290 Trier, Germany

**Keywords:** Computational neuroscience, Neural circuits

## Abstract

Restless legs syndrome (RLS) in pregnancy is a common disorder with a multifactorial etiology. A neurological and obstetrical cohort of 308 postpartum women was screened for RLS within 1 to 6 days of childbirth and 12 weeks postpartum. Of the 308 young mothers, 57 (prevalence rate 19%) were identified as having been affected by RLS symptoms in the recently completed pregnancy. Structural and functional MRI was obtained from 25 of these 57 participants. A multivariate two-window algorithm was employed to systematically chart the relationship between brain structures and phenotypical predictors of RLS. A decreased volume of the parietal, orbitofrontal and frontal areas shortly after delivery was found to be linked to persistent RLS symptoms up to 12 weeks postpartum, the symptoms' severity and intensity in the most recent pregnancy, and a history of RLS in previous pregnancies. The same negative relationship was observed between brain volume and not being married, not receiving any iron supplement and higher numbers of stressful life events. High cortisol levels, being married and receiving iron supplements, on the other hand, were found to be associated with increased volumes in the bilateral striatum. Investigating RLS symptoms in pregnancy within a brain-phenotype framework may help shed light on the heterogeneity of the condition.

## Introduction

Restless legs syndrome (RLS) is a common sensorimotor disorder with a 5–10% prevalence in the adult population^1^. Clinically, this condition is characterized by (1) an urge, triggered by unpleasant sensations, to move the legs, (2) momentary relief following movement, (3) worsening of the sensations with rest, and (4) a tendency for the sensations to occur in the evening or at night. With two recognized forms, primary (idiopathic) and secondary, RLS (in both of its forms) is considered to be a continuous spectrum with a genetic contribution at one end and an environmental or comorbid disease contribution at the other^2^. While idiopathic (primary) RLS is thought to be strongly influenced by genetic predisposition^3^, the secondary form of the condition is linked to neurodegenerative disorders, diseases affecting the peripheral nervous system (e.g. diabetic neuropathy, amyloid neuropathy^4^), metabolic disorders, iron deficiency, anemia and diabetes^2^. Another common condition, in which RLS symptoms frequently manifest for the first time or worsen, is pregnancy^5^. Unlike its idiopatic form, pregrancy-related RLS is largerly a transitory condition. Hovewer, like gestational diabetes or hypertension, the first manifestation of RLS symptoms during pregnacy is frequently an early indicator of the condition’s potential recurrence later in life^6^.

RLS symptoms triggered by pregnancy tend to increase from 0% before pregnancy to 23% in the third trimester^7, 8^. Experience of RLS in a previous pregnancy and family history of idiopathic RLS are strong predictors of the new manifestation of RLS symptoms during the ongoing pregnancy^6^. Along with the genetic predisposition, physiological adaptations during pregnancy contribute crucially to the manifestation of RLS^8^. Changes such as the dramatic increases in estrogen and progesterone levels^9^, insulin resistance^10^, hypervolemia^9^, weight gain^11^ and increased iron use^12^ can render pregnant women particularly susceptible to the development of RLS symptoms. In addition, transient RLS during pregnancy is a risk factor for the chronic idiopathic form^6^. Thus, pregnancy-related RLS cannot be clearly distinguished from its idiopatic counterpart^13^.

The diagnosis of RLS in pregnancy (analogous to idiopathic RLS) is based on the criteria developed by the International Restless Legs Syndrome Study Group (IRLSSG), according to which RLS is characterized by a number of neurological and other clinical indicators including the severity and frequency of repetitive compulsive movements as well as sleep and mood disturbances^14^. Pregnancy-related RLS symptoms are not only a neurological condition, they also represent an obstetrical one with likely links to gestational diabetes mellitus^2^ or pregnancy-induced hypertension^15^. Thus, the manifestation of RLS in pregnancy may herald the risk of cardiovascular and metabolic diseases. However, the available data in this respect are inconclusive. In addition, multiparity^16^, anemia and age (RLS is seen more frequently in older women)^17–19^ have been suggested to be further phenotypical predictors of RLS in pregnancy. Finally, RLS frequently co-occurs with psychiatric disorders, particularly affective disorders^20^. In the context of pregnancy and childbirth, pre-pregnancy RLS has been found to be linked to perinatal depression^21^.

In sum, RLS in pregnancy is a sensorimotor neurological disturbance with significant obstetrical and environmental contributions, causing not only physical but also substantial psychological distress^22^. To understand this condition, the complex interaction of clinical, environmental and anamnestic aspects needs to be taken into account. In a large neurological and obstetrical sample of 308 early-postpartum women, we sought to investigate a number of possible neurological, obstetrical and anamnestic phenotypical predictors of RLS in pregnancy (e.g. history of RLS in previous pregnancies, persistence of the RLS symptoms after childbirth (up to 12 weeks postpartum), clinical character of the symptoms, socioeconomic characteristics (age, income), pregnancy-related complications (gestational diabetes, hypertension), iron supplements, stressful life events (SLE), depressivity, multiparity, duration of pregnancy, birth of a twin/or singleton pregnancy, and child’s weight). Adding a brain structure analysis, we assessed the phenotypical predictors of RLS in pregnancy in a brain-phenotype model, seeking to explain how RLS patients differ from one another. An innovative multivariate pattern-learning algorithm (involving canonical correlation analysis or CCA) was employed to conjointly characterize patterns of brain structure and the phenotypical predictors of RLS, facilitating an objective assessment of their complex interplay.

## Results

### Course of RLS in pregnancy

57 (19%) of the 308 participants reported to experience RLS symptoms during pregnancy. While for 22 (39%) of them this was the first pregnancy, for 35 (61%), it was a second or third pregnancy. Of these 35 women, 20 (57%) had experienced RLS symptoms during a previous pregnancy. 19 (33%) of the 57 women continued to experience RLS symptoms 12 weeks after delivery. In this subgroup of participants with persisting RLS symptoms, the severity of the symptoms was lower 12 weeks after delivery (T1) (M = 15.68, SD = 7.22) compared to the symptom severity at T0 (M = 18.95, SD = 5.54), t(18) = 3.314, *p* = 0.04. For 2 (4%) women, no information was available with respect to RLS symptoms 12 weeks postpartum. In 38 participants (67%), the RLS symptoms were reported to have been most severe during the third trimester of pregnancy (for details, see Table 1).

**Table 1:** Differences between women with and without RLS.

	RLS (*n* = 57)	No RLS (*n* = 251)	*p*
	M (SD)	M (SD)	
Age	33.18 (4.66)	31.47 (4.608)	.012
EPDS score T0	4.72 (4.02)	5.01 (3.53)	.583
EPDS score T1	3.77 (3.23)	4.00 (4.03)	.690
HCC T0 (in pg/mg)	11.45 (16.14)	9.36 (11.54)	.260
HCC T1 (in pg/mg)	5.97 (5.95)	13.65 (95.70)	.549
Hemoglobin in g/dL T0	12.06 (1.13)	11.94 (1.18)	.483
Days of gestation	272.65 (9.96)	272.66 (13.50)	.997
Birthweight of child (in gram)	3315.69 (501.96)	3326.28 (571.94)	.902

	*n* (%)	*n* (%)	
Unmarried	21 (36.8)	60 (24)	.047
Total number of children			.186
1	26 (45.6)	137 (55)	
2	21 (36.8)	86 (34.5)	
> 3	10 (17.6)	26 (10.4)	
Highest degree of education			.511
No school graduation	1 (1.7)	2 (0.8)	
Secondary school degree	3 (5.3)	8 (3.3)	
Junior high school degree	5 (8.8)	38 (15.6)	
University entrance	48 (84.2)	195 (80.3)	
Birth mode			.808
Spontaneous birth	31 (54.4)	142 (56.6)	
Ventouse birth	4 (7)	15 (6)	
Planned C-section	13 (22.8)	65 (25.9)	
Emergency C-section	9 (15.8)	29 (11.6)	
Breastfeeding at T0	49 (86)	221 (88)	.666
Pregnancy-related complications			
Pre-eclampsia/Hypertension	4 (7.0)	13 (5.2)	.583
Gestational Diabetes	11 (19.3)	29 (11.6)	.049
Relocation of child to other ward	69 (27.6)	17 (29.8)	.603
Iron supplements	32 (58.2)	16 (61.5)	.690
RLS in previous pregnancy	20 (35.0)	–	
Most severe RLS symptoms^1^			
First trimester	2 (3.51)	–	
Second trimester	7 (12.28)	–	
Third trimester	38 (66.67)	–	
Equally high over all trimesters	8 (14.04)	–	
Psychiatric history	11 (19.3)	41 (16.4)	.599
Baby blues	30 (52.6)	108 (43.7)	.223
At least one stressful life event	35 (61.4)	103 (41.4)	.006

Notes. *n* = absolute frequency, M = mean, SD = standard deviation; EPDS = Edinburg Postnatal Depression Scale; HCC = Hair cortisol concentration; T0 = baseline measure in childbed; T1 = 12 weeks postpartum.

^**1**^Participant were asked the following question: “At which point during your pregnancy did you experience the RLS symptoms as most frequent or severe?”.

### Differences between RLS and control groups

Participants with RLS symptoms during pregnancy were older (Mdn = 35) compared to their counterparts without RLS (Mdn = 31), U = 8.688, *p* = 0.12. Also, women in the RLS group had more often gestational diabetes (n = 10, 17.5%) compared to those in the control group without RLS (n = 29, 11.6%), χ^2^ (1, 308) = 6.040, *p* = 0.049. In addition, a significantly larger number of women in the RLS group (n = 35, 61.4%) experienced SLE compared to the control group (n = 103, 41.4%), χ^2^ (1, 306) = 7.522, *p* = 0.006 and were not married, χ^2^ (1, 307) = 3.941, = 0.047 (see Table 1). On the other hand, the groups with and without RLS did not differ with respect to depressivity (based on the Edinburgh Postnatal Attachment Scale, EPDS^23^) and hair cortisol concentration (HCC) at any time point and hemoglobin in g/dL at T0. Also, there were no group differences in terms of severity of baby blues, birth mode, multiparity and psychiatric history.

### Structural differences between RLS and age-matched control group

The structural MRI data of RLS patients and age-matched healthy controls were analyzed using voxel-based morphometry (VBM) analysis. No significant between-group differences were identified in an independent t-test using the cluster-correction threshold combined with a liberal voxel-wise threshold (*p* < 0.01).

### Resting-state differences between RLS and age-matched control group

The resting-state fMRI (rsfMRI) data of RLS patients were analyzed in relation to those of the age-matched healthy controls. No significant group differences were identified in an independent t-test for any of the voxel-wise or region-based rsfMRI measures using the cluster-correction threshold combined with a liberal voxel-wise threshold (*p* < 0.01).

### Detecting behavioral descriptors that distinguish patients with RLS

**Figure 1:**
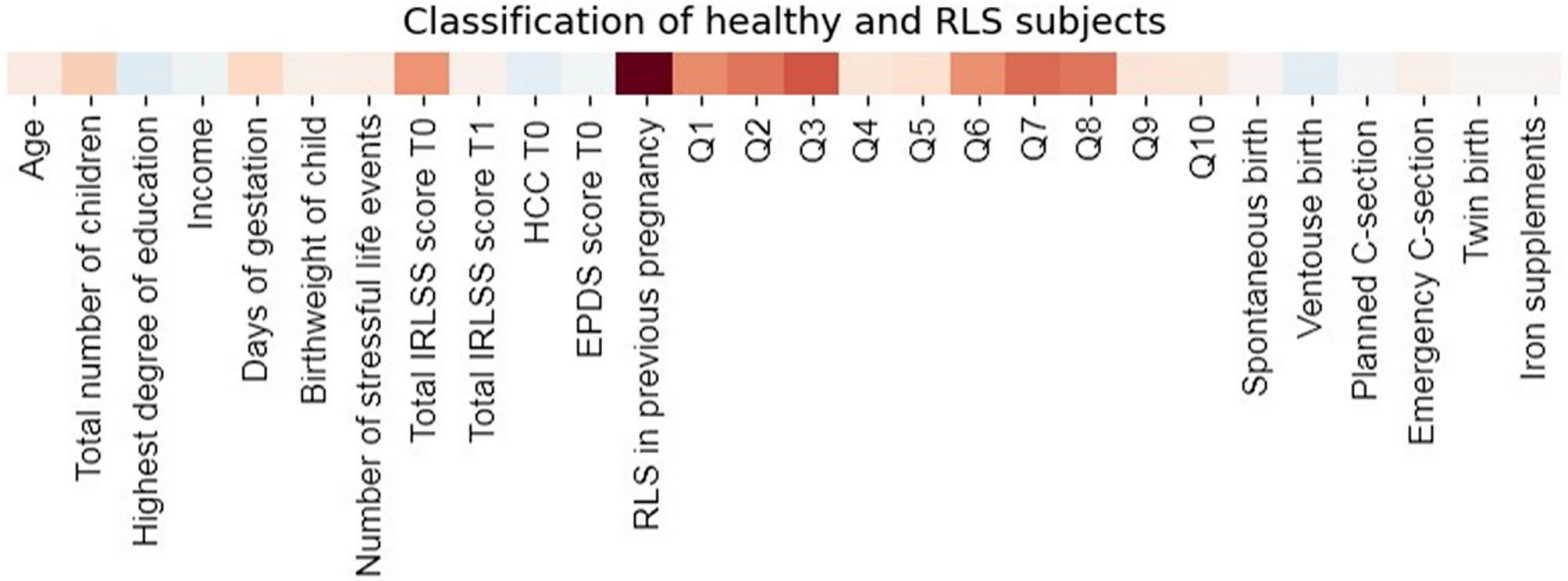
Informative features for predicting RLS. * IRLSS score T0 = Severity of RLS symptoms in pregnancy; IRLSS score T1 = Severity of RLS symptoms 12 weeks postpartum. A modern constrained version of logistic regression was deployed to explore the contribution of each anamnestic and clinical indicator to the prediction of RLS experience. The x-axis depicts the variables included in the analysis. The red square indicates the feature contributing to the detection of an RLS patient while the blue square indicates the feature responsible for detecting a healthy participant. For example, a high score in the feature “RLS in previous pregnancy” would tip the balance of the output toward being an RLS patient. In sum, the most informative features with respect to the prediction of RLS were RLS in a previous pregnancy, and a high score against the IRLSS items 1, 2, 3, 6, 7 and 8 at T0. See Table 2.

In a group of 57 RLS patients and 26 matched healthy controls, we explored the contribution of each anamnestic and clinical indicator to the detection of patients with RLS. Eight out of 28 variables were highly weighted for detecting patients with RLS. Apart from the total and IRLSS, other features indicative of RLS were experience of RLS in a previous pregnancy, and a high score with respect to the questions 1, 2, 3, 6, 7 and 8 (Fig. 1, Table 2). Here, the items describing the degree of RLS symptom relief by exercise (Q3), the urge to move the legs (Q2) and the average severity of the unpleasant sensations in the legs (Q8) were most indicative of patients with RLS. On the other hand, items describing the degree of sleep disturbance (Q4, Q5) as well as being affected in the daily activities (Q9) or mood disturbances (Q10) were the least indicative. Age, number of children, duration of pregnancy, birthweight or mode of birth also did not contribute to the detection of participants affected by RLS in pregnancy.

**Table 2:** Demographics of women with RLS and their matched counterparts, in accordance with Fig. 1

	RLS (*n* = 57)			Matched women without RLS (*n* = 26)		
	Min	Max	M (SD)	Min	Max	M (SD)
Age	23	42	33.18 (4.66)	24	40	32.12 (4.13)
Total number of children	1	5	1.80 (0.97)	1	3	1.54 (0.58)
Highest degree of education^1^	0	3	2.65 (0.79)	1	3	2.85 (0.64)
Income^2^	1	4	3.42 (0.74)	2	4	3.54 (0.71)
Days of gestation	239	288	272.65 (9.96)	238	287	273 (12.39)
Birthweight of child (gram)	2130	4290	3315.69 (501.96)	2090	4395	3417.69 (518.13)
Number of stressful life events	0	7	1.44 (1.76)	0	2	0.31 (0.62)
Total IRLSS total score T0	5	36	17.95 (7.28)			
Total IRLSS total score T1	0	29	5.42 (8.60)			
HCC T0	1.33	77.84	11.45 (16.14)	1.52	73.95	11.59 (14.98)
EPDS score T0	0	19	4.72 (4.02)	0	9	4.27 (2.65)

	*n* (%)			*n* (%)		
RLS in previous pregnancy	20 (35.1)			0		

	Min	Max	M (SD)			
IRLSS—Q1^3^	1	4	2.24 (0.96)			
IRLSS—Q2^4^	1	4	2.56 (0.96)			
IRLSS—Q3^5^	0	3	1.75 (0.80)			
IRLSS—Q4^6^	0	4	1.87 (1.44)			
IRLSS—Q5^7^	0	4	1.36 (1.24)			
IRLSS—Q6^8^	1	4	2 (0.82)			
IRLSS—Q7^9^	1	4	2.53 (0.94)			
IRLSS—Q8^10^	1	4	2 (0.77)			
IRLSS—Q9^11^	0	3	0.64 (0.83)			
IRLSS—Q10^12^	0	4	1 (1.25)			

	*n* (%)			*n* (%)		
Spontaneous birth	31 (54.4)			19 (73.1)		
Ventouse birth	4 (7.0)			1 (3.8)		
Planned C-section	13 (22.8)			5 (19.2)		
Emergency C-section	9 (15.8)			1 (3.8)		
Twin birth	6 (10.5)			1 (3.8)		
Iron supplements	32 (56.1)			16 (61.5)		

Notes: *n* = absolute frequency, % = relative frequency, M = mean, SD = standard deviation, Md = median, Min = Minimum, Max = Maximum, IRLSS = International Restless Legs Syndrome Scale, SLESQ = Stressful Life Events Screening Questionnaire; EPDS = Edinburgh Postnatal Depression Scale, HCC = hair cortisol concentration, T0 = baseline measure in childbed; T1 = 12 weeks postpartum.

^1^None (0), secondary school (1), junior high school degree (2), university entrance (3).

^2^In k euros/year; < 10 k (1), < 20 k (2), < 50 k (3), > 50 k (4).

^3^ Q1 (T0): How would you rate the RLS-related unpleasant sensations in your legs or arms?.

^4^ Q2 (T0): How would you rate your urge to move because of the unpleasant sensations?.

^5^ Q 3 (T0): To what extent were the unpleasant sensations in your legs or arms alleviated by exercise?.

^6^ Q4 (T0): How badly was your sleep affected by the unpleasant sensations?.

^7^ Q5 (T0): How tired or sleepy were you during the day because of the unpleasant sensations?.

^8^ Q6 (T0): Overall, how strong were the unpleasant sensations?.

^9^ Q7 (T0): How often did the unpleasant sensations occur?.

^10^ Q8 (T0): If you had unpleasant sensations, how severe were they on average?.

^11^ Q9 (T0): To what extent did your unpleasant sensations affect your ability to carry out daily activities?.

^12^ Q10 (T0): How badly was your mood affected by the unpleasant sensations (making you, for example, angry, depressed, sad, anxious or irritable)?.

### First CCA analysis: coherent morphological patterns across a whole brain atlas

**Figure 2:**
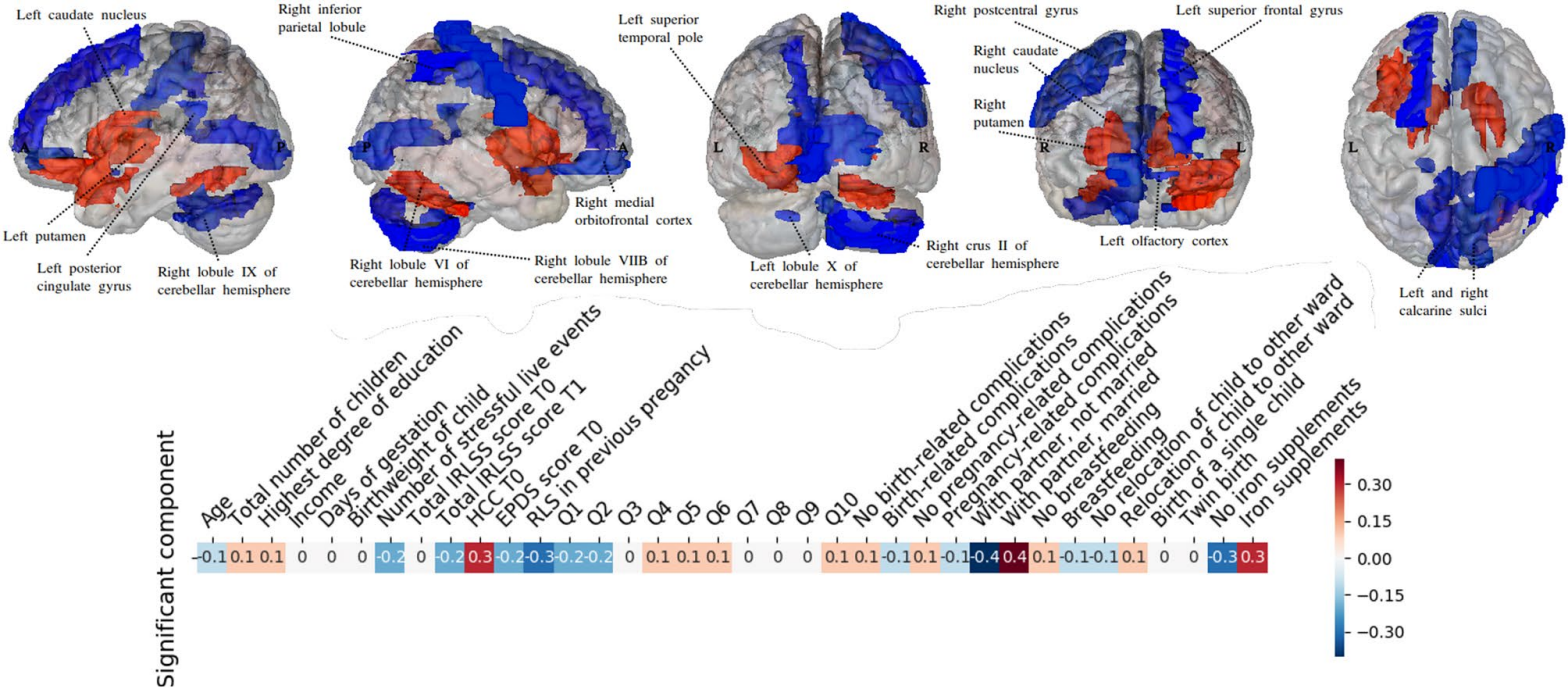
CCA reveals multivariate patterns of link between phenotypical predictors of RLS and brain structure. * IRLSS score T0 = Severity of RLS symptoms in pregnancy; IRLSS score T1 = Severity of RLS symptoms 12 weeks postpartum. The figure shows the first canonical model of brain-behavior co-variation that was statistically significant based on permutation testing (*p* < 0.05). In the top row, the brain structure loadings are plotted from the left, right, posterior, anterior and superior views. The blue regions indicate the negative CCA loading associated with volume in this specific region, while the red region volumes indicate a positive CCA loading for this specific region. The bottom row depicts the loadings of the patient’s anamnestic and clinical indicators (blue for a negative loading and red for a positive one). A high hair cortisol level (HCC) during pregnancy, being married, and receiving iron supplement were robustly linked to an increased size of the left superior temporal pole, the left inferior frontal gyrus, the basal ganglia (right and left caudal nucleus and putamen), and the right lobule 6 of the cerebellar hemisphere. On the other hand, RLS during previous pregnancy (positive history of RLS), not being married and not receiving any iron supplement were found to be linked to a decreased volume size of the right inferior parietal lobule, the left posterior cingulate gyrus, the right lobule 9, 8B, crus 2 and the left lobule 10 of the cerebellar hemisphere, the left olfactory cortex, the left superior frontal gyrus, the right postcentral gyrus, the left and right calcarine sulci and the right medial orbitofrontal cortex. Further anamnestic and clinical changes located at the negative end of the mode included complaining during pregnancy about the severity of RLS-related sensations (Q1 of IRLSS score at T0), the degree of the urge to move the legs (Q2 of IRLSS score at T0), having RLS symptoms at 12 weeks postpartum (IRLSS score at T1) and a higher SLE number. That the most inter-hemispherically coherent brain-phenotype associations were found in the basal ganglia justified our targeted analysis (Fig. 1, Table 3).

The main objective of the whole-brain study was to identify components of the relationship between brain structures and clinical traits that described RLS patients in pregnancy. A single statistically significant CCA mode emerged in the first analysis (r = 0.993, *p* < 0.05), exhibiting a strong covariation of brain volume measures and diverse anamnestic and clinical indicators (Fig. 2, Table 3). The other 9 of the 10 estimated modes were not significantly robust. The most important CCA mode comprised measures that varied along a positive–negative axis. As regards the anamnestic and clinical indicators, a high hair cortisol (HCC) level, being married, and receiving iron supplements were located at the positive end of the mode. The brain regions at the positive end of the mode included the left superior temporal pole, the left inferior frontal gyrus, the basal ganglia (right and left caudate nucleus and putamen), and the right lobule 6 of the cerebellar hemisphere. Note that only the basal ganglia changes were located in both hemispheres. The most significant anamnestic and clinical changes located at the negative end of the mode included positive history of RLS in previous pregnancy, not being married and not receiving any iron supplement. Further anamnestic and clinical changes located at the negative end of the mode included complaining during pregnancy about the severity of RLS-related sensations (Q1), the degree of the urge to move the legs (Q2) during pregnancy, the SLE number and persisting RLS symptoms 12 weeks postpartum as indicated by IRLSS score at T1. Brain regions with high negative loadings included the right inferior parietal lobule, the left posterior cingulate gyrus, the right lobule 9, VIIIb, crus II and the left lobule 10 of the cerebellar hemisphere, the left olfactory cortex, the left superior frontal gyrus, the right postcentral gyrus, the left and right calcarine sulci and the right medial orbitofrontal cortex. In sum, our results identified several inter-hemispheric zones of activity, especially in the basal ganglia, contributing to coherent anamnestic and clinical changes. Based on this observation, we focused on this particular region in a second analysis.

**Table 3:** Demographics of women with RLS participated on MRI, in accordance with Figs. 2 and 3.

	Descriptive statistics		
	Min	Max	M (SD)
Age	23	42	33.08 (5.84)
Total number of children	1	5	2.04 (1.23)

	Min	Max	Mode
Highest degree of education^1^	0	3	3
Income^2^	1	4	3

	Min	Max	M (SD)
Days of gestation	239	288	274 (10.97)
Birth weight of child (gram)	2700	6300	3704 (847.77)
Number of stressful life events	0	6	1.2 (1.56)
IRLSS total score T0	7	28	16.87 (6.26)
IRLSS total score T1	0	29	7.2 (9.82)
HCC T0	1.32	77.84	9.2 (15.38)
EPDS score T0	0	11	4.37 (3.46)
RLS in previous pregnancy	0	2	1.12 (0.79)
IRLSS—Q1^3^	1	3	2.08 (0.77)
IRLSS—Q2^4^	1	4	2.37 (0.92)
IRLSS—Q3^5^	1	3	1.71 (0.75)
IRLSS—Q4^6^	0	4	1.96 (1.43)
IRLSS—Q5^7^	0	4	1.37 (1.13)
IRLSS—Q6^8^	1	3	1.96 (0.69)
IRLSS—Q7^9^	0	4	2.42 (0.93)
IRLSS—Q8^10^	0	2	0.46 (0.58)
IRLSS—Q9^11^	0	2	0.46 (0.58)
IRLSS—Q10^12^	0	3	0.71 (0.91)

	N (%)		
Birth-related complications	8 (32.00)		
Pregnancy-related complications	14 (56.00)		
With partner, married	13 (52.00)		
Breastfeeding	19 (76.00)		
Relocation of child to other ward	7 (28.00)		
Birth of a single child	21 (84.00)		
Twin birth	4 (16.00)		
Iron supplements	16 (64.00)		

### Second CCA analysis: exploratory voxel-level investigation within the basal ganglia

**Figure 3:**
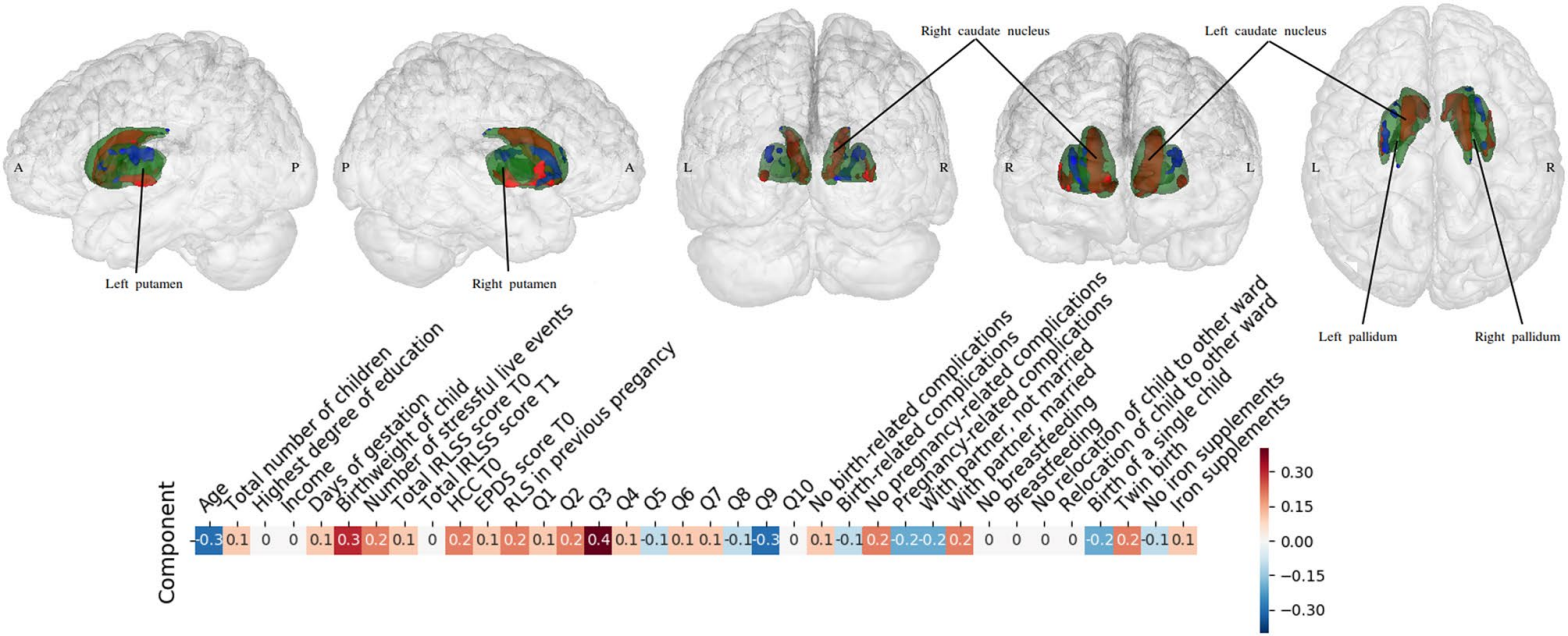
Specific patterns linking the basal ganglia to the phenotypical predictors of RLS. * IRLSS score T0 = Severity of RLS symptoms in pregnancy; IRLSS score T1 = Severity of RLS symptoms 12 weeks postpartum. A targeted post-hoc CCA was applied to find coherent associations between the basal ganglia volume changes and changes in the patients’ anamnestic and clinical indicators. While the above-mentioned analysis (Fig. 1) considered the whole brain as measured by volume estimates, the present analysis focused on the left and right basal ganglia at a more fine-grained voxel resolution. Green indicates the anatomy of the target regions as part of the basal ganglia. In the top row, the loadings of the basal ganglia voxels are plotted from the left, right, posterior, anterior and superior views. The green area contours expose the outer shape of the basal ganglia voxels, which cover the striatum (putamen and caudate nucleus) and the pallidum. Within the basal ganglia, the blue area indicates negative CCA loadings associated with those specific voxels while the red indicates positive CCA loadings for those specific voxels. The bottom row depicts the loadings of the patient’s behavioral and clinical indicators (blue for negative and red for positive). While having a baby with higher birth weight and having during pregnancy the unpleasant sensations in the legs or arms alleviated by exercise (Q3) were strongly linked to an increased size of the body and head of the left and right caudate nuclei and the left and right inferior putamen, being young and not being so affected by RLS to carry out daily activities (Q9) were found to be associated with a decreased size of the superior part of the left and right putamen. In sum, our analysis revealed strong associations between anamnestic changes and changes in the medial and lateral striatum within the basal ganglia. See Table 3**.**

As a logical follow-up, we studied the relationship between changes in the phenotypical predictors and specific changes within the basal ganglia in RLS patients in pregnancy (Fig. 3, Table 3). To that end, the first 10 principal components of standardized brain volume of the basal ganglia regions and the first 10 principal components of standardized anamnestic and clinical data were fed into the CCA, with only one CCA mode being computed given the previous results. The anamnestic and clinical changes located at the positive end of the mode included having a heavier baby and having the unpleasant sensations in the legs or arms alleviated by exercise (Q3), while the basal ganglia changes at the positive end of the mode were the left and right body and head of the caudate nucleus and the left and right inferior putamen. The amnestic and clinical changes at the negative end of the mode were being young and not being so affected by RLS on a daily basis (Q9), while the corresponding brain volume changes included the superior part of the putamen.

In short, major changes were found in the basal ganglia, with an inter-hemispheric increase in the size of the caudate and the inferior part of the putamen as the size of the baby and RLS-related sleep deprivation increased.

## Discussion

In a large neurological and obstetrical sample of 308 postpartum women, participants with RLS during pregnancy were identified and compared with the rest of the sample. Additionally, a number of clinical and anamnestic indicators of RLS in pregnancy were assessed by leveraging state-of-the-art machine learning algorithms. Within the group of women with RLS, the phenotypical predictors of the condition were assessed by means of a brain structure analysis of the brain-phenotype model. The group of young mothers with RLS symptoms during recent pregnancy was compared with a control group (postpartum women without any RLS symptoms).

The RLS prevalence rate in our sample was found to be 19%. While in 30% of the cases the symptoms were still reported 12 weeks postpartum, 70% of the women experienced a complete recovery of RLS symptoms shortly after delivery. The majority of participants (about 70% of women with RLS) experienced the RLS symptoms as most severe and most frequent in the last trimester of pregnancy. In line with previous findings^24, 25^, those afflicted by RLS in our sample were older and more often affected by gestational diabetes than their unaffected counterparts. Women experiencing RLS in pregnancy were also found to have experienced SLE more often than those without RLS^26^. Additionally, there were group differences in terms of marital status, with women in the RLS group being more often not married. Examining the possible predictive factors of RLS in pregnancy, we found a history of RLS related to a previous pregnancy to be the strongest predictor of the condition in recent pregnancy, which is in line with previous reports^6^. In IRLSS, items describing the degree of urge to move the legs and the degree of subsequent symptom relief were better predictors of RLS symptoms compared to those describing daily activity, mood or sleep.

### Neuroimaging data: group comparison based on brain structure and connectivity

We did not find any difference in brain structure and function between RLS patients and controls. With respect to brain structure, our results demonstrate that there are no consistent gray matter alterations to constitute a reliable surrogate neuroimaging marker for idiopathic RLS. The results of a recent meta-analysis^27^ point to a similar conclusion. This coordinate-based meta-analysis (12 voxel-based morphometry studies, 375 RLS subjects and 385 healthy controls) could not identify any evidence of consistent gray matter alterations in idiopathic RLS. According to the authors, this lack of consistency may be attributed to differences in sample size, genetics, gender distribution and age at onset, and clinical heterogeneity (e.g. clinical course, disease severity or disease duration)^27^. Another meta-analysis, based on resting-state studies (7 studies, 134 RLS subjects and 142 healthy controls), demonstrated differential functional connectivity in the thalamic and dopaminergic pathways in idiopathic RLS^28^. We, however, did not find any group differences in functional connectivity, which may be due to the fact that the RLS symptoms in pregnancy are largely transitory. The neuroimaging data were obtained shortly after childbirth with most of the women already experiencing relief or partial mitigation of the symptoms. While we did not seek to quantify CNS iron deposits by means of MRI, some studies suggest that the assessment of brain iron through quantitative magnetic susceptibility measurement reveals differences between healthy controls and RLS patients in the basal ganglia and the dentate nucleus^29, 30^. However, further investigations are needed to determine if regional brain iron concentrations are consistently lower in patients with RLS^31^.

### RLS group: relationship between whole-brain gray matter morphology and phenotypical predictors

As regards the relationship between whole-brain gray matter morphology and phenotypical predictors within the group of women affected by RLS in pregnancy, high HCC reflecting cumulative cortisol exposure during the last trimester, being married and receiving iron supplements were found to be associated with increased volumes of the left superior temporal pole, the left inferior frontal gyrus and the basal ganglia (the right and left caudate nucleus and putamen). Previous studies have foreshadowed a possible physiological link between factors such as the circadian rhythm of cortisol secretion or iron deficiencies in the central nervous system (CNS) and manifestation of idiopathic RLS. In particular, an evening and early night hour RLS symptom increase has been suggested to be moderated by low-dose hydrocortisone^32, 33^. Apart from the circadian rhythm of cortisol secretion, reduction of brain iron is thought to be one of the risk factors in the development of RLS symptoms^32^, the iron deficiency being attributed to dysregulations in the dopamine system^34^. Also noteworthy is the relationship seen between marital status and increased basal ganglia structures in RLS patients. Married individuals are suggested to be healthier^35, 36^ and therefore less likely to be exposed to stressors compared to those who either never married or were previously married^37^. We suggest that the positive relationship between brain volume in the striatum and frontal areas and such factors as higher HCC reflecting the cumulative cortisol exposure during the last trimester, iron supplementation or being married are likely to be associated with more favorable prognoses with respect to RLS symptoms. At the same time, persistent symptoms beyond pregnancy (up to 12 weeks postpartum), a history of RLS in previous pregnancy, the severity of RLS-related sensations and the intensity of the urge to move the legs in the latest pregnancy were found to be associated with a decreased volume size of the (right inferior) parietal lobule, the posterior cingulate, and the right medial orbitofrontal and frontal (left superior frontal gyrus) cortices. The same negative relationship was observed between brain volume in the above-mentioned regions and not being married, not receiving any iron supplement, higher SLE numbers and higher EPDS score shortly after childbirth. Based on these observations, we suggest that the association between reduced brain volume and factors such as history of RLS in previous pregnancy, not being married, higher SLE numbers, and being more strongly and persistently affected by RLS symptoms in the latest pregnancy is probably indicative of a less favorable prognosis for RLS. This assumption is supported by the fact that a history of RLS in previous pregnancies and the intensity of the felt urge to move the legs in the latest pregnancy were found to be the strongest predictors of the RLS group (as compared to the group unaffected by RLS) in our sample. Previous research has indicated links between SLE, such as child maltreatment, and the development of a set of somatic and visceral central sensitivity syndromes like chronic pain, irritable bowel syndrome as well as RLS^26^.

### Limitations and summary

As for the limitations of the study, there are a few. First, the diagnosis of RLS was based solely on a self-report questionnaire (IRLSS), although it is a widely used measure in RLS diagnosis with a good level of content validity^14^. Second, as the symptoms of RLS were assessed retrospectively, the risk of recall bias cannot be completely ruled out. Third, we did not have any information regarding the onset of RLS symptoms in participants who experienced persistent RLS symptoms at 12 weeks postpartum. Thus, we could not ascertain whether the RLS symptoms in this subgroup of women predated the latest pregnancy or had been triggered by it. Finally, we did not perform any neurological examination to control for conditions potentially mimicking RLS symptoms (e.g. periodic limb movements during sleep (PLMS), painful leg and moving toes syndrome or generalized restlessness similar to akathisia^38^) and did not monitor if the symptoms were present during the MRI scanning. Although, given the good level of IRLSS validity, the RLS-mimicking conditions were unlikely in our sample. In spite of these limitations, to our knowledge, ours is the first study to apply multimodal neuroimaging (structural and functional MRI) to investigate pregnancy-related RLS symptoms. These are of course preliminary results; the analysis needs to be conducted with a larger group of participants. However, our study is the first to identify the components (indicators) that need to be considered when assessing this issue in a larger simple. Our results also show that a number of phenotypical predictors of RLS symptoms during pregnancy are linked to the striatal and frontoparietal structures. The most important and interhemispheric variations occur in the basal ganglia, with the medial and lateral striatum contributing the most to the clinical and anamnestic differences in individuals experiencing RLS symptoms in pregnancy. Along with our results, the human lesion studies^39–41^ as well as the recent RLS animal model studies^42^ underscore the crucial role of the basal ganglia in the manifestation of the RLS spectrum. Based on the brain-phenotype model, we conclude therefore that different factors such as severity and history of RLS symptoms, stress response, history of SLE or life circumstances (e.g. marital status) or iron deficiencies in the CNS contribute to the manifestation of RLS in pregnancy. Interconnected in a complex way, these factors, along with genetic predisposition, likely lead to the dysfunction of the mesolimbic and nigrostriatal dopaminergic pathways, triggering abnormalities in the limbic/nociceptive and sensorimotor networks^32^ and the development of RLS symptoms. The investigation of RLS in pregnancy in a brain-phenotype model has the potential of helping augment our understanding of the heterogeneity of the spectrum.

## Methods

### Study participants and clinical and neuropsychological assessments

**Figure 4:**
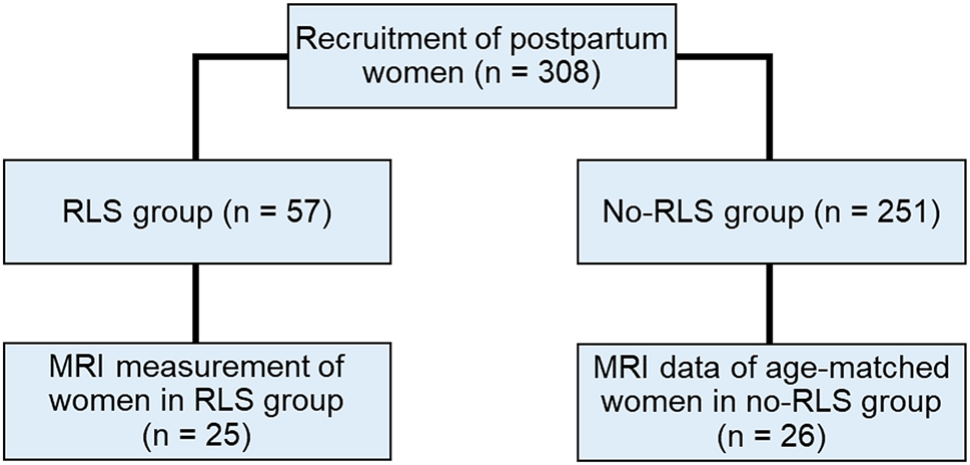
Flowchart of inclusion into the study.

This study is part of an ongoing longitudinal project (risk of postpartal depression, RIPOD study) aiming at early recognition of postpartum depression (PPD). A total of 308 women were recruited in the Department of Gynecology and Obstetrics at the University Hospital Aachen within 1 to 6 days of childbirth (T0) (Fig. 4) between January 2016 and April 2018. All women eligible for the MRI (n = 108), underwent an MRI experiment within 1 to 6 days postpartum. Following receipt of the participants’ informed consent, all participants were retrospectively screened for RLS symptoms in pregnancy by means of the International Restless Legs Syndrome Rating Scale (IRLSS)^14^. The participants were instructed to fill up the IRLSS at T0 based on the time frame when they are affected the most. In addition, they were required to answer the following questions: “At which point during your pregnancy did you experience the RLS symptoms as most frequent or severe?” (having to choose from the following answers: “1st trimester (03.-12. week of pregnancy); 2nd trimester (13.-24. week of pregnancy); 3rd trimester (25.-40. week of pregnancy); Similarly across all trimesters; I’ve had no symptoms”) and “At which point in time did your RLS symptoms disappear?” (with the option to indicate that the RLS symptoms were still present).

The exclusion criteria were alcoholic or psychotropic substance dependency or use during pregnancy, anti-depressive or anti-psychotic medication during pregnancy, history of psychosis or manic episodes, depressive episode at the time of inclusion into the study, and inadequate proficiency in German or English. To control for the exclusion criteria, a brief non-standardized clinical interview was conducted according to the DSM V criteria by an experienced psychiatrist (NC). A detailed description of the study population is given in Tables 1 and 2.

Based on the multimodal neuroimaging data (n = 108) collected at T0 (within 1 to 6 days postpartum), 25 participants had RLS symptoms during the recent pregnancy (aged 23–42 years). The remainder of the RLS group (n = 32) was not eligible for the MRI. Neuroimaging data of the age-matched control group without RLS during pregnancy were collected from the same data pool. A detailed description of the sample that participated in the MRI experiment is given in Table 3 (please refer also to Fig. 4 with the flowchart of inclusion into the study).

In addition to the IRLSS, the following questionnaires were used at T0: the Stressful Life Events Screening Questionnaire (SLESQ)^43^ was used as a self-report instrument to help assess the participants' encounter with 13 particularly traumatic experiences, and the Edinburgh Postnatal Depression Scale (EPDS)^23^ was used as a self-report instrument for the screening of postpartum depression. Furthermore, the participants filled out a standardized questionnaire to help obtain anamnestic and pregnancy-related information, e.g. family history of psychiatric conditions, previous psychiatric history, income, marital status, complications during pregnancy or birth mode.

The second screening session for postpartum RLS symptoms took place 12 weeks after delivery (T1) when the IRLSS and EPDS were applied for the second time.

Hair samples were collected shortly after delivery (T0) and 12 weeks postpartum (T1) to assess the cumulative cortisol exposure over the last trimester of pregnancy and three months postpartum, respectively.

In our sample, oral supplementation of 200–300 mg / day was given from Hb < 10.5 based on the German maternity guidelines.

The study was conducted in compliance with the Helsinki Declaration and was approved by the local ethics committee of the Medical Faculty, RWTH Aachen University, Germany.

### Hair sample collection and preparation

Hair cortisol is widely regarded as biomarkers of chronic stress^44^. Hair samples were obtained from the posterior vertex of the head, stored in aluminum foil, and treated as described in^45^. 3 cm of hair was cut at each time point and approximately 50 mg was weighed in a polypropylene sampling tube using a microbalance. Hair samples were washed with 2-propanol, extracted with a fourfold deuterium isotope-labeled internal standard of cortisol and methanol for 24 h and then analyzed with liquid chromatography triple quadrupole mass spectrometry using an ion trap (Agilent Technologies 1200 infinity series -QTRAP 5500 ABSciex). The limits of quantification were 0.05 ng/mL or 2 pg/mg hair, respectively.

### MRI procedure and Voxel-based morphometry

The structural image was acquired with an anatomical 3D T1-weighted MPRAGE sequence (176 slices, TR = 2300 ms, TE = 1.99 ms, FoV = 256 × 256 mm^2^, flip angle = 9°, voxel resolution = 1 × 1 × 1 mm^3^). The brain tissue was segmented into gray matter, white matter and cerebrospinal fluid, the resultant adjusted volume measurements representing the amount of gray matter corrected for individual brain size. Structural MRI data were preprocessed using the Computational Anatomy Toolbox (CAT12) and SPM12 (https://www.fil.ion.ucl.ac.uk/spm/software/spm12/) toolbox implemented in Matlab 2015b (MathWorks, Inc., Natick, MA) to derive voxel-wise gray matter volumes for each subject. The default settings of CAT12 were applied for spatial registration, normalization and segmentation of the T1-weighted structural brain images. For a precise spatial normalization into standard (MNI), the Diffeomorphic Anatomic Registration Through Exponentiated Linear algebra algorithm (DARTEL)^46^ was performed. The images were segmented into gray matter, white matter, and cerebrospinal fluid, and modulated with Jacobian determinants. Finally, the modulated gray matter images were smoothed with an 8 mm isotropic FWHM Gaussian kernel. Subsequently, the association between brain structure and clinical indicators of patients experiencing RLS in pregnancy was systematically charted.

Data of 25 women with RLS during pregnancy and n = 26 age-matched women without RLS during pregnancy, were analyzed using SPM12 toolbox. Smoothed gray matter segment of all participant were implemented in an independent t-test analysis, using the theory of Gaussian random fields. Total intracranial volume (TIV) was entered as control variables into the model. Although the groups were age-matched, age was used as an additional control variable in all GMV analyses. To avoid edge effects at the border between different tissues, an absolute masking with a threshold of < 0.1 was applied^47^. All contrasts were evaluated for significance using an exact permutation-based cluster threshold (1000 permutations permuting group labels) (*p* < 0.05) combined with an uncorrected voxel-threshold of *p* < 0.01.

### Assessing the clinical and anamnestic indicators in predicting RLS

Independent samples t-tests (for continuous variables) and chi-square tests (for categorical variables) were used to assess differences between the RLS and control groups. Within-group differences were analyzed by means of paired samples t-tests, with IBM Statistics 25 (SPSS, Chicago, IL) being used for the analysis.

The relative importance of the clinical and anamnestic indicators for predicting RLS was analyzed using a L2-penalized logistic regression based on the information of 57 RLS patients and 26 matched healthy controls. The L2 regularization was used to reduce the chances of overfitting, which can render the models’ prediction of future observation unreliable^48^. The L2-penalized logistic regression estimated the separating hyperplane (i.e., a linear function), distinguishing between patients with RLS and healthy participants. The outcome to be predicted was defined by being healthy (0) or being a patient with RLS (1). The model parameters were then fit to optimally predict RLS based on all the standardized clinical and anamnestic data.

### Resting-state functional connectivity (RSFC)

For preprocessing of resting-state data of women with RLS during pregnancy and n = 26 age-matched women without RLS during pregnancy, the SPM12 software was used, implemented in Matlab 2015b (MathWorks, Inc., Natick, MA). Images were realigned, unwarped, and co-registered to the structural image, spatially normalized using structural information, and smoothed by a Gaussian convolution with 6 mm FWHM. A gray matter (GM) mask was applied to reduce all analyses to gray matter tissue. Images were further processed in the CONN toolbox version 18.b^49^. White matter (WM), cerebrospinal fluid (CSF) and 24 motion parameters (Friston-24) were added as first-level covariates when computing voxel- and region-based measures of interest. Global Correlation was calculated as the average of bivariate correlations between the BOLD signal of a given voxel and every other voxel^49^. Integrated Local Correlation was computed as the average bivariate correlation between each voxel and its neighboring voxels weighted by a Gaussian convolution with 6 mm FWHM^50^. Fractional Amplitude of Low Frequency Fluctuations (fALFF) was calculated at each voxel as the root mean square of the BOLD signal amplitude in the analysis frequency band (here 0.01 – 0.08 Hz) divided by the amplitude in the entire frequency band^51^.

#### Voxel-based analyses

 Voxel-wise group comparisons were performed in SPM12 using independent t-tests and age as a covariate. All contrasts were evaluated for significance using an exact permutation-based cluster threshold (1000 permutations permuting group labels) (*p* < 0.05) combined with an uncorrected voxel-threshold of *p* < 0.01.

### Brain-phenotype association across the whole-brain gray matter

The relationship between brain structure and clinical and anamnestic indicators of patients experiencing restless legs syndrome (RLS) in pregnancy was systematically charted based on the structural magnetic resonance imaging (T1-MRI) data from RLS patients in pregnancy.

### Signal extraction

Using the Automated Anatomical Labeling (AAL) ROI atlas^52^, quantitative measures of gray matter volume were extracted within the 116 macroscopic brain structures labeled in this atlas in every patient. Widely used in neuroimaging^53–55^, the AAL atlas is the result of an automated anatomical parcellation of the spatially normalized single-subject high-resolution scan of the brain.

For the extraction of relevant signal from the structural brain data, the total of 116 regions served as topographic masks to average the volume information across the voxels belonging to a given region. Each AAL region was represented by the average gray matter volume across all AAL region voxels. This way of engineering the morphological brain features yielded as many volumetric brain variables per patient as the total number of AAL regions (i.e., 116). All region-wise structural volumes were transformed into z-scores by mean centering and unit-variance scaling^56^.

### Joint multivariate decomposition across the whole brain

A multivariate pattern analysis technique, namely the canonical correlation analysis (CCA), was employed^57^. This approach relates multiple blocks of data (with structural imaging features on the one hand, and anamnestic and clinical indicators on the other) through a latent factor model. Projections of the first 10 principal components of the standardized volumetric brain measures (i.e., 25 * 10 matrix) as well as the first 10 principal component projections of the standardized anamnestic/clinical data (i.e., 25 * 10 matrix) were fed into a CCA algorithm, the number 25 indicating the number of subjects included in the analysis. This multivariate and multimodality approach enabled us to objectively assess the relationship between patient differences in brain volume and anamnestic/clinical indicators. The CCA determines the canonical vectors u and v that maximize the symmetric relationship between a linear combination of brain volumes (X) and a linear combination of anamnestic and clinical data (Y), thereby identifying the two projections, Xu and Yv, that yield maximal linear co-occurrence between sets of regions with volumetric changes and sets of patients’ clinical changes. In concrete terms, the positive (negative) modulation weights revealed increased (decreased) strengths of brain-behavior association relative to the baseline volumetric changes.

In sum, using the CCA, which finds linear combinations to optimize correlations between brain region volumes and an array of anamnestic/clinical measures, we estimated pairs of canonical variates along which sets of anamnestic/clinical measures and patterns of brain volume correlated coherently across subjects. Henceforth, we refer to each pair of such variates as a ‘mode’ of coherent brain-phenotype co-variation.

### Testing for statistically significant brain-behavior associations between brain volume and phenotypical indicators

Following the CCA of RLS patients, the statistical robustness of the ensuing brain-phenotype relationship was assessed through a non-parametric permutation approach^58, 59^ using the canonical correlation as the test statistic. Relying on minimal modeling assumptions, a valid null distribution was derived for the achieved correlation between the canonical variates resulting from the CCA analysis. In 1,000 permutation iterations, the brain volume matrix was held constant, while the anamnestic and clinical data matrix underwent patient-wise random shuffling. While the constructed surrogate data preserved the statistical structure idiosyncratic to the MRI-derived signals, they were permitted to selectively destroy the signal property related to the CCA statistic to be tested^57^. The empirical distribution thus generated reflected the null hypothesis of random association between volume and anamnestic and clinical indicators across patients. The Pearson correlation r between the perturbed canonical variates were recorded in each iteration, with the *p* values being obtained from the number of correlations r from the null CCA model. This analysis revealed a single significant CCA mode relating brain volume to the subjects’ clinical and anamnestic measures (*p* < 0.05).

### Post-hoc brain-phenotype association of the basal ganglia

With the whole-brain results showing the basal ganglia to be the site of the most important and inter-hemispheric variations across brain volumes, we further investigated patterns of changes in the basal ganglia specifically related to the anamnestic and clinical indicators. Quantitative measures of gray matter volume were extracted within the basal ganglia brain structures, the left and right caudate and putamen, as labeled in AAL in every participant. This way of engineering morphological brain features yielded as many volumetric brain variables per patient as the total number of voxels included in these AAL regions (i.e., 10,881). All region-wise structural volumes were transformed into z-scores by mean centering and unit-variance scaling. The first 10 principal components of the standardized basal ganglia volumetric brain measures as well as the first 10 principal components of the standardized clinical and anamnestic data were fed into a CCA. Following the preceding whole-brain CCA (cf. previous paragraph), only the first mode was automatically computed.

## Data Availability

Python was selected as the scientific computing engine, the open-source ecosystem of which helps enhance replicability, reusability and provenance tracking. Scikit-learn^60^ provided efficient, unit-tested implementations of state-of-the-art statistical learning algorithms (http://scikit-learn.org). All analysis scripts of the present study are readily accessible to the reader online (https://github.com/JLefortBesnard/RLS_2019).
